# Theoretical accuracy for indirect predictions based on SNP effects from single-step GBLUP

**DOI:** 10.1186/s12711-022-00752-4

**Published:** 2022-09-27

**Authors:** Andre Garcia, Ignacio Aguilar, Andres Legarra, Shogo Tsuruta, Ignacy Misztal, Daniela Lourenco

**Affiliations:** 1 Department of Animal and Dairy Science, University of Georgia, Athens, GA 30602 USA; 2 Instituto Nacional de Investigación Agropecuaria (INIA), 11500 Montevideo, Uruguay; 3 UMR GenPhySE, INRA Toulouse, BP52626, 31326 Castanet Tolosan, France

## Abstract

**Background:**

Although single-step GBLUP (ssGBLUP) is an animal model, SNP effects can be backsolved from genomic estimated breeding values (GEBV). Predicted SNP effects allow to compute indirect prediction (IP) per individual as the sum of the SNP effects multiplied by its gene content, which is helpful when the number of genotyped animals is large, for genotyped animals not in the official evaluations, and when interim evaluations are needed. Typically, IP are obtained for new batches of genotyped individuals, all of them young and without phenotypes. Individual (theoretical) accuracies for IP are rarely reported, but they are nevertheless of interest. Our first objective was to present equations to compute individual accuracy of IP, based on prediction error covariance (PEC) of SNP effects, and in turn, are obtained from PEC of GEBV in ssGBLUP. The second objective was to test the algorithm for proven and young (APY) in PEC computations. With large datasets, it is impossible to handle the full PEC matrix, thus the third objective was to examine the minimum number of genotyped animals needed in PEC computations to achieve IP accuracies that are equivalent to GEBV accuracies.

**Results:**

Correlations between GEBV and IP for the validation animals using SNP effects from ssGBLUP evaluations were ≥ 0.99. When all available genotyped animals were used for PEC computations, correlations between GEBV and IP accuracy were ≥ 0.99. In addition, IP accuracies were compatible with GEBV accuracies either with direct inversion of the genomic relationship matrix (**G**) or using the algorithm for proven and young (APY) to obtain the inverse of **G**. As the number of genotyped animals included in the PEC computations decreased from around 55,000 to 15,000, correlations were still ≥ 0.96, but IP accuracies were biased downwards.

**Conclusions:**

Theoretical accuracy of indirect prediction can be successfully obtained by computing SNP PEC out of GEBV PEC from ssGBLUP equations using direct or APY **G** inverse. It is possible to reduce the number of genotyped animals in PEC computations, but accuracies may be underestimated. Further research is needed to approximate SNP PEC from ssGBLUP to limit the computational requirements with many genotyped animals.

## Background

One of the ways to deal with the ever-increasing number of genotyped animals in single-step genomic best linear unbiased prediction (ssGBLUP) evaluations is to include only animals with valuable information (own and progeny records) in the evaluations, and then compute indirect predictions (IP) for the remaining young, genotyped animals [1–3]. In future evaluations, when these animals (for instance, young heifers) have a record or progeny, they could be considered in ssGBLUP; and if they are culled, they would not be considered in ssGBLUP. In addition, IP can be a useful tool to provide fast, interim evaluations for young, genotyped animals, and can also serve as a genomic prediction for animals not included in official evaluations (for instance, genotypes sent from foreign countries). Such predictions reduce the time necessary between collecting a DNA sample and getting predictions on young animals, which allows farmers and artificial insemination (AI) studs to make faster management decisions and thus to reduce rearing costs by culling animals earlier [4, 5]. When genomic BLUP (GBLUP) or ssGBLUP is used for genomic evaluations, effects of single nucleotide polymorphisms (SNPs) are not readily available but they can be easily backsolved from genomic estimated breeding values (GEBV) using formulas as shown by VanRaden [6], Strandén and Garrick [7] and Wang et al. [8]. Once SNP effects are calculated, IP are obtained for young animals as the sum of the SNP effects weighted by the gene content. Most national dairy cattle genomic predictions use this procedure to run periodical evaluations, obtain estimates of SNP effects (although usually by multi-step procedures), and release fast interim predictions based on IP. In the following, and to avoid confusion, we will call GEBV the direct estimate of the genomic breeding value obtained through ssGBLUP, whereas we use the name IP for the estimate of the genomic breeding value obtained as the sum of SNP effects weighted by the gene content.

Typically, in animal breeding programs, the accuracy of predicted breeding values is calculated to help make selection decisions. Henderson [9] showed that accuracies of EBV can be obtained based on the prediction error variance (PEV), and the latter may be obtained by directly inverting the coefficient matrix of the BLUP mixed model equations (MME). When the system of equations becomes too big, it is impossible to invert the coefficient matrix to obtain PEV even with current computing resources. To overcome this limitation, approximations have been proposed and implemented for pedigree-based evaluations [10] and when genomic information is included [11–15]. Thus, the problem of calculating genomic accuracies of GEBV obtained through GBLUP or ssGBLUP has already been addressed. Similarly, it is interesting for producers to have a measure of the accuracies of IP, to make early selection decisions with more confidence.

Strandén and Christensen [16] showed how to calculate accuracies for IP based on the prediction error covariance (PEC) of SNP effects. These authors and, Tier et al. [17] as well, pointed out that the reliability of GEBV depends on allele coding, and that by back-solving SNP PEC from the same model (ssGBLUP) the accuracy of both GEBV and IP are correctly aligned.

Liu et al. [12] explained that the cost of obtaining such reliabilities from SNP-BLUP is smaller because the size of the left-hand side (LHS) of the MME depends mainly on the number of SNPs rather than the number of genotyped animals. Because of the equivalence between SNP-BLUP and GBLUP, it is also possible to obtain the PEC for SNP effects when using (ss)GBLUP. However, for (ss)GBLUP, the computational cost depends on the number of genotyped animals. Derivations to obtain SNP PEC under the (ss)GBLUP model were described by Gualdron Duarte et al. [18] and Aguilar et al. [19]. In principle, exact computation of SNP PEC requires that the whole matrix of PEC across all genotyped animals is obtained from the inverse of the MME. This can be very costly in time and memory.

Pocrnic et al. [14] investigated the accuracy of genomic selection under a GBLUP model using the algorithm for proven and young (APY) and showed that when only a small number of eigenvalues from the genomic relationship matrix (GRM) was used, it was sufficient to account for a large portion of the genetic variation. Because the dimensionality of the genomic information is limited [20, 21], it is possible to reduce the number of animals needed to calculate SNP effects and IP [2, 22]. Likewise, the limited dimensionality could also allow for a reduction in the number of animals needed to obtain SNP PEC and accuracies for IP under (ss)GBLUP.

The objectives of this study were to: (1) present equations to compute individual accuracy of IP by back-solving PEC of GEBV from ssGBLUP into PEC of SNP effects, and to investigate the feasibility of this method; (2) test the algorithm for APY in PEC computations; (3) investigate the minimum number of genotyped animals for which the complete PEC matrix needs to be computed, to obtain IP accuracy in large genotyped populations.

## Methods

### Data and model

Data for this study were provided by the American Angus Association (Saint Joseph, MO) and included 230,639 animals in the pedigree and 38,000 post-weaning gain (PWG) phenotypes. Genotypes for 39,774 SNPs after quality control, were available for 60,000 animals born up to 2018. To mimic a real situation, genotyped animals were split into “old” (N = 54,533) and “validation” (N = 5467), i.e., young, genotyped animals predicted through IP. Validation animals were genotyped animals that were born in 2018 and for which their pedigree, progeny, and own records were omitted from the data. Validation animals were first excluded from ssGBLUP, and IP and their accuracies obtained; then, these IP accuracies were compared to accuracies when these same validation animals were included in ssGBLUP.

The statistical model for PWG was $\mathbf{y}=\mathbf{X}\mathbf{b}+\mathbf{W}\mathbf{u}+\mathbf{e}$, where $\mathbf{y}$ is a vector of post-weaning gain phenotypes and $\mathbf{b}$ is a vector of fixed contemporary group effects; $\mathbf{u}$ is the vector of random additive genetic effects, and $\mathbf{e}$ is the vector of random residuals; $\mathbf{X}$ and $\mathbf{W}$ are the incidence matrices relating $\mathbf{y}$ with the effects in $\mathbf{b}$ and $\mathbf{u}$, respectively. Genomic evaluations were implemented using single-step GBLUP.

In ssGBLUP, the inverse of the relationship matrix that combines pedigree and genomic information $({\mathbf{H}}^{-1})$ was constructed as in Aguilar et al. [23]:

1 $${\mathbf{H}}^{-1}={\mathbf{A}}^{-1}+\left[\begin{array}{cc}{\mathbf{0}}& {\mathbf{0}}\\ {\mathbf{0}}& {\mathbf{G}}^{-1}-{\mathbf{A}}_{\bf{22}}^{-1}\end{array}\right],$$

where ${\mathbf{G}}^{\bf{-1}}$ is the inverse of the genomic relationship matrix, ${\mathbf{A}}^{\bf{-1}}$ and ${\mathbf{A}}_{\bf{22}}^{\bf{-1}}$ are the inverses of the pedigree relationship matrix for all and genotyped animals, respectively. All three matrices considered inbreeding. The initial genomic relationship matrix was constructed as the type 1 matrix in VanRaden [6]:

2 $${\mathbf{G}}_{0}=\frac{\mathbf{ZZ}^{\mathbf{{\prime}}}}{2\sum {\text{p}}_{i}(1-{\text{p}}_{i})},$$

where $\mathbf{Z}$ is a matrix of gene content centered for twice the allele frequency of SNP $i$ (${\text{p}}_{i}$). Allele frequencies were calculated based on the current genotyped population and recalculated for each evaluation. In this study, $\mathbf{G}$ was constructed as:

3 $$\mathbf{G}=b\left(\left(1-\alpha \right){\mathbf{G}}_{0}+\alpha {\mathbf{A}}_{22}\right)+{\bf{11}}^{\mathbf{\prime}}\delta ,$$

where $\alpha$ = 0.05 is a blending parameter [6], and $\delta$ and $b$ are tuning parameters calculated as in Vitezica et al. [24]:

4 $$\delta =\frac{1}{{\text{n}}^{2}}\left(\sum_{i}\sum_{j}{\mathbf{A}}_{22 i,j}-\sum_{i}\sum_{j}{\mathbf{G}}_{i,j}\right) \text{and}\; b=1-\frac{1}{2}\delta .$$

After tuning and blending steps, $\mathbf{G}$ is invertible and compatible with the pedigree relationships.

Since for large-scale genomic evaluations, it becomes unfeasible to directly invert $\mathbf{G}$, the algorithm for proven and young (APY) was proposed by Misztal et al. [25] and Misztal [26] to overcome this limitation. In APY, the genotyped animals are divided into core (c) and non-core (n) animals:

5 $$\mathbf{G}=\left[\begin{array}{cc}{\mathbf{G}}_{\text{cc}}& {\mathbf{G}}_{\text{cn}}\\ {\mathbf{G}}_{\text{nc}}& {\mathbf{G}}_{\text{nn}}\end{array}\right].$$

And ${\mathbf{G}}_{\text{APY}}^{-1}$ is calculated as follows:

6 $${\mathbf{G}}_{\text{APY}}^{-1}=\left[\begin{array}{cc}{\mathbf{G}}_{\text{cc}}^{-1}& {\bf{0}}\\ {\bf{0}}& {\bf{0}}\end{array}\right]+\left[\begin{array}{c}-{\mathbf{G}}_{\text{cc}}^{-1}{\mathbf{G}}_{\text{cn}}\\ \mathbf{I}\end{array}\right]{\mathbf{M}}_{\text{nn}}^{-1}\left[-{\mathbf{G}}_{\text{nc}}{\mathbf{G}}_{\text{cc}}^{-1}\boldsymbol{ }\mathbf{I}\right].$$

With elements of ${\mathbf{M}}_{\text{nn}}$, the Mendelian error diagonal matrix, obtained for the $i$ *th* non-core animal as:

7 $${\text{m}}_{\text{nn},i}={\text{g}}_{ii}-{\mathbf{G}}_{i\text{c}}{\mathbf{G}}_{\text{cc}}^{-1}{\mathbf{G}}_{\text{c}i}.$$

The number of core animals in APY can be obtained as the number of largest eigenvalues explaining 98 to 99% of the variance in $\mathbf{G}$, which can be found by the eigenvalue decomposition of $\mathbf{G}$ or the singular value decomposition of $\mathbf{Z}$ [21]. In this study, the number of eigenvalues explaining 98% and 99% of the variance in $\mathbf{G}$ was 11,413 and 15,242, respectively; therefore 15,000 core animals were randomly selected to be used in APY.

Once ${\mathbf{H}}^{-1}$ is built, the ssGBLUP MME for PWG are:

8 $$\left[ {\begin{array}{*{20}{c}} {{\bf{X'}}{{\bf{R}}^{ - 1}}{\bf{X}}}&{{\bf{X'}}{{\bf{R}}^{ - 1}}{\bf{W}}}\\ {{\bf{W'}}{{\bf{R}}^{ - 1}}{\bf{X}}}&{{\bf{W'}}{{\bf{R}}^{ - 1}}{\bf{W}} + {{\bf{H}}^{ - 1}}\sigma _{\text{u}}^{ - 2}} \end{array}} \right]\left[ {\begin{array}{*{20}{c}} {\hat {\varvec{\upbeta}} }\\ {\hat {\bf{u}}} \end{array}} \right]{\text{ = }}\left[ {\begin{array}{*{20}{c}} {{\bf{X'}}{{\bf{R}}^{ - 1}}{\bf{y}}}\\ {{\bf{W'}}{{\bf{R}}^{ - 1}}{\bf{y}}} \end{array}} \right],$$

where $\mathbf{R}=\mathbf{I}{\sigma }_{\text{e}}^{2}$ is the residual variance and ${\sigma}_{\text{u}}^{2}$ the additive genetic variance; $\widehat{\varvec{\upbeta}}$ and $\widehat{\mathbf{u}}$ are the estimates of fixed effects and GEBV, respectively.

### Benchmark GEBV and accuracy

A ssGBLUP evaluation using the complete data (i.e., including validation animals) was run to obtain benchmark GEBV accuracy (${\text{ACC}}_{\text{GEBV}}$) for validation animals. The ${\text{ACC}}_{\text{GEBV}}$ for animal $j$ was calculated based on PEV from the inverse of the LHS of MME (8) as follows:

Let the inverse of the coefficient matrix of the MME (8) be:

9 $${\mathbf{C}}^{-1}=\left[\begin{array}{cc}{\mathbf{C}}^{{\varvec{\upbeta}}{\varvec{\upbeta}}}& {\mathbf{C}}^{{\varvec{\upbeta}}\mathbf{u}}\\ {\mathbf{C}}^{{\bf{\upbeta}}\mathbf{u}}& {\mathbf{C}}^{\mathbf{u}\mathbf{u}}\end{array}\right].$$

10 $$\text{Then}, {\text{ACC}}_{{\text{GEBV}}_{j}}\text{=}\sqrt{{1}- \, \frac{{\text{PEV}}_{\text{j}}}{{\sigma}_{\text{u}}^{2}}},$$

where ${\text{PEV}}_{\text{j}}$ is the diagonal element $j$ in the prediction error variance matrix ${\mathbf{C}}^{\mathbf{u}\mathbf{u}}$.

### Indirect predictions and accuracy

Before calculating IP, SNP effects from ssGBLUP were obtained as described in Wang et al. [8], using the POSTGSF90 program [27]. Recently, Legarra et al. [28] showed that under ssGBLUP, blending and tuning parameters need to be taken into account when back-solving GEBV into SNP effects ($\mathbf{a}$):

11 $$\widehat {\mathbf{a}}|\widehat {\mathbf{u}} = \left( {1 - \alpha } \right){{b}}\frac{1}{{2\sum {{{\text{p}}_i}} \left( {1 - {{\text{p}}_i}} \right)}}{\mathbf{Z'}}{{\mathbf{G}}^{ - {\bf{1}}}}\widehat {\mathbf{u}},$$

where $\widehat{\mathbf{u}}$ is a vector of GEBV from an ssGBLUP evaluation with the reduced data that does not include data for validation animals. Once SNP effects are available, IP can be calculated as $\mathbf{IP } = {\mathbf{Z}}_{\text{validation}}\widehat{\mathbf{a}}$, which reflects the marker-based predictions [28].

Liu et al. [12] showed how to compute accuracies for IP from a SNP-BLUP model using SNP PEC as follows. Let the inverse coefficient matrix of the SNP-BLUP MME be:

12 $${\bf{C}}_{\bf{g}}^{ - 1} = \left[ {\begin{array}{*{20}{c}} {{{\bf{C}}^{{\varvec{\upbeta\upbeta }}}}}{{{\bf{C}}^{{\varvec{\upbeta g}}}}}\\ {{{\bf{C}}^{{\varvec{\upbeta g}}}}}{{{\bf{C}}^{{\bf{gg}}}}} \end{array}} \right].$$

13 $${\text{Then, }{\text{ACC}}}_{{\text{IP}}_{j}}=\sqrt{{1}-\frac{{\text{PEV}}_{j}}{{\sigma}_{\text{u}}^{2}} \, },$$

where ${\text{ACC}}_{{\text{IP}}_{j}}$ is the accuracy of IP for animal $j$; ${\text{PEV}}_{\text{j}} = {{\text{z}}}_{\text{j}}{\mathbf{C}}_{\mathbf{g}}^{\mathbf{gg}}{\text{z}}^{\prime}_{\text{j}}$; ${\mathbf{C}}^{\text{gg}}$ is the SNP PEC matrix and ${\mathbf{z}}_{j}$ is the row vector from the $\mathbf{Z}$ matrix, that contains the centered genotypes for animal $j$. Since SNP-BLUP and GBLUP are equivalent models, SNP PEC from SNP-BLUP (or ss-SNP-BLUP) or from (ss)GBLUP are the same. Gualdron Duarte et al. [18] and Aguilar et al. [19], showed that PEC of SNP effects can be calculated as follows:

14 $${\text{var}}\left( {\widehat {\bf{a}}} \right) = {\text{PEC}} = {\text{var}}\left( {\left( {1 - \alpha } \right)b\frac{1}{{2\sum {{{\text{p}}_i}} \left( {1 - {{\text{p}}_i}} \right)}}{\bf{Z'}}{{\bf{G}}^{ - {\bf{1}}}}{{\widehat {\mathbf{u}}}}} \right)$$

15 $$= \left( {1 - \alpha } \right){\rm{b}}\frac{1}{{2\Sigma {{\rm{p}}_i}\left( {1 - {{\rm{p}}_i}} \right)}}{\bf{Z'}}{{\bf{G}}^{ - {\bf{1}}}}{\rm{var}}\left( {\widehat {\bf{u}}} \right)\left( {\left( {1 - \alpha } \right){\rm{b}}\frac{1}{{2\Sigma {{\rm{p}}_i}\left( {1 - {{\rm{p}}_i}} \right)}}{\bf{Z'}}{{\bf{G}}^{ - {\bf{1}}}}} \right),$$

16 $$= \left( {1 - \alpha } \right){\rm{b}}\frac{1}{{2\sum {{{\rm{p}}_i}} \left( {1 - {{\rm{p}}_i}} \right)}}{\bf{Z'}}{{\bf{G}}^{ - {\bf{1}}}}\left( {{\rm{var}}\left( {\bf{u}} \right) - {\rm{var}}\left( {\widehat {\bf{u}} - {\bf{u}}} \right)} \right){{\bf{G}}^{ - {\bf{1}}}}{\bf{Z}}\frac{1}{{2\sum {{{\rm{p}}_i}} \left( {1 - {{\rm{p}}_i}} \right)}}{\rm{b}}\left( {1 - \alpha } \right).$$

Then,

17 $${\text{var}}\left( {\widehat {\mathbf{a}}} \right) = {\text{PEC}} = \left( {1 - \alpha } \right){{b}}\frac{1}{{2\sum {{{\text{p}}_i}} \left( {1 - {{\text{p}}_i}} \right)}}{\bf{Z'}}{{\mathbf{G}}^{ -{\bf{ 1}}}}\left( {{\mathbf{G}}\sigma _{\text{u}}^2 - {{\mathbf{C}}^{{{\text{u}}_2}{{\text{u}}_2}}}} \right){{\mathbf{G}}^{ - {\bf{1}}}}{\mathbf{Z}}\frac{1}{{2\sum {{{\text{p}}_i}} \left( {1 - {{\text{p}}_i}} \right)}}b\left( {1 - \alpha } \right).$$

Therefore,

18 $${\text{var}}\left( {\widehat {\mathbf{a}}} \right)={\text{PEC}}=\left( {1 - \alpha } \right){{b}}\frac{1}{{2\sum {{{\text{p}}_i}} \left( {1 - {{\text{p}}_i}} \right)}}({\bf{Z'}}{{\mathbf{G}}^{ - {\bf{1}}}}{\bf{Z}}\sigma _{\mathbf{u}}^2 - {\bf{Z'}}{{\mathbf{G}}^{ - {\bf{1}}}}{{\mathbf{C}}^{{{\mathbf{u}}_2}{{\mathbf{u}}_2}}}{{\mathbf{G}}^{ - {\bf{1}}}}\ {\mathbf{Z}})\frac{1}{{2\sum {{{\text{p}}_i}} \left( {1 - {{\text{p}}_i}} \right)}}b\left( {1 - \alpha } \right).$$

Note that $\alpha$ and $b$ are blending and tuning parameters, accounted for in PEC computations, and ${\mathbf{C}}^{{\text{u}}_{2}{\text{u}}_{2}}$ is part of the inverse of the LHS of MME (8) corresponding to genotyped animals.

Once SNP PEC is available, accuracy for IP for animal $j$ (${\text{ACC}}_{{\text{IP}}_{j}}$) can be computed as:

19 $${\rm{AC}}{{\rm{C}}_{{\rm{I}}{{\rm{P}}_j}}} = \sqrt {1 - \frac{{\left( {1 - \alpha } \right)b\;{\bf{z}}{}_j{\rm{var}}\left( {\widehat {\bf{a}}} \right){{{\bf{z'}}}_{{j}}}}}{{\sigma _{\rm{u}}^{\rm{2}}}}} .$$

While the accuracy of IP can be easily obtained with small datasets, obtaining ${\mathbf{C}}^{{\text{u}}_{2}{\text{u}}_{2}}$ becomes impractical in large-scale evaluations because of the number of genotyped animals. To overcome this limitation, the dimensionality of genomic information was exploited by using the APY algorithm to compute a sparser ${\textbf{G}}^{-{\bf{1}}}$. Lourenco et al. [22] and Garcia et al. [2] showed that correlations between IP obtained based on SNP effects from all genotyped animals or only core animals from APY under ssGBLUP were higher than 0.98, with greatly reduced computing cost when using only core animals.

### Implementation

The implementation required changes in three existing programs from the BLUPF90 software suite [27]. Most of the changes followed those for the computation of p-values for SNP effects as described in [19]. In a nutshell, the modifications in BLUPF90 allows storing the inverse of the LHS of the ssGBLUP MME in binary format. For that, OPTION snp_var is required. When this option is also used in POSTGSF90, the program reads the binary file, extracts the coefficients for genotyped animals (${\mathbf{C}}^{{\text{u}}_{2}{\text{u}}_{2}}$), and applies Eq. (18). The main difference compared to the calculation of p-values is that POSTGSF90 saves all the elements of the SNP PEC matrix in binary format for further computations of ${\text{ACC}}_{\text{IP}}$ by PREDF90, whereas only SNP PEV (i.e., the diagonal of the SNP PEC matrix) are needed in POSTGSF90 for the computation of p-values of SNP effects as shown in [19]. PREDF90, which is a software to compute IP, was then modified to read PEC from a file and compute the accuracy of IP based on Eq. (19). For obtaining accuracy of IP in PREDF90, the argument –acc has to be used.

### Feasibility and validation of IP accuracies

Three main scenarios were designed to test the computations of IP accuracies from ssGBLUP. In the first scenario (*direct*; S1), all data were used in ssGBLUP, except for the validation animals; therefore, the number of genotyped animals in ssGBLUP was 54,533. In the second scenario (*apy*; S2), we tested the feasibility of using APY ${\textbf{G}}^{-{\bf{1}}}$ in the PEC computations; therefore, APY ${\textbf{G}}^{-{\bf{1}}}$ replaced ${\textbf{G}}^{-{\bf{1}}}$ in ssGBLUP. Finally, in the third scenario (S3), we investigated the possibility of reducing the number of genotyped animals to decrease the cost of computing PEC. This scenario was subdivided by using different numbers of animals in the calculation of PEC (S3.x). In S3.1 (*50K-2K*), different sets of genotyped animals were randomly selected (50K, 40K, 30K, 20K, 10K, 5K, or 2K). For scenarios S3.2 to S3.5, 15K genotyped animals were selected based on different criteria: core animals in S3.2 (*core*); genotyped animals with a high accuracy in S3.3 (*hacc*); core animals plus their progeny phenotypes in S3.4 (*core_prog*); and high accuracy animals plus their progeny phenotypes S3.5 (*hacc_prog*). More details on all the scenarios are provided below:

(S1) *direct*: all genotyped animals (N = 54,533) and phenotypes with direct ${\textbf{G}}^{-{\bf{1}}}$;

(S2) *apy*: all genotyped animals (N = 54,533) and phenotypes with APY ${\textbf{G}}^{-{\bf{1}}}$;

(S3.1) 5*0K-2K*: all phenotypes and decreasing the number of genotyped animals from 50K to 2K;

(S3.2) *core*: genotypes for core animals only (N = 15K) and all phenotypes;

(S3.3) *hacc*: genotypes for high accuracy animals only (N = 15K) and all phenotypes;

(S3.4) *core_prog*: genotypes and phenotypes for core animals plus their progeny phenotypes;

(S3.5) *hacc_prog*: genotypes and phenotypes for high accuracy animals plus their progeny phenotypes.

The scenarios S1 and S2 (*direct* and *apy*) used all the data available, and they reflected the case when all animals in the evaluation are used to calculate SNP PEC_._ These scenarios also served as a test to compare the direct or APY ${\textbf{G}}^{-{\bf{1}}}$ in the PEC computations. The other scenarios (under S3) represented a situation when only a subset of the animals is used. In scenario S3.3 (*hacc*), 15,000 animals with the highest accuracy based on the benchmark (${\text{ACC}}_{\text{GEBV}}$) were selected. The number of animals with genotypes, phenotypes, and pedigree for each scenario and dataset is in Table 1. Once SNP PEC were available, ${\text{ACC}}_{\text{IP}}$ was calculated, in PREDF90, for validation animals in each scenario and dataset. Regardless of the number of animals used to obtain PEC in each scenario, GEBV used to backsolve SNP effects were always obtained from the first scenario (i.e., direct), including “old” animals only, thus mimicking a real situation where GEBV are available from the complete official evaluation. The ${\text{ACC}}_{\text{IP}}$ for the validation animals, computed from all scenarios, were compared with the benchmark ${\text{ACC}}_{\text{GEBV}}$ calculated when the validation animals were included in the ssGBLUP evaluation (as in Eq. (9)).

**Table 1:** Number of animals with genotypes, phenotypes, and pedigree information in each scenario

Scenario	Genotypes	Phenotypes	Pedigree
direct	54,533	38,000	230,639
apy	54,533	38,000	230,639
2K-50K	2K-50K	38,000	230,639
core (15K)	15,000	38,000	230,639
hacc (15K)	15,000	38,000	230,639
core_prog (15K)	15,000	22,625	101,837
hacc_prog (15K)	15,000	32,673	106,051

*direct*: all genotyped animals (N = 54,533) and phenotypes with direct ${\textbf{G}}^{-{\bf{1}}}$; *apy*: all genotyped animals (N = 54,533) and phenotypes with APY ${\textbf{G}}^{-{\bf{1}}}$; *50K-2K*: all phenotypes and decreasing the number of genotyped animals from 50K to 2K; *core*: genotypes for core animals only (N = 15K) and all phenotypes; *hacc*: genotypes for high accuracy animals only (N = 15K) and all phenotypes; *core_prog*: genotypes and phenotypes for core animals plus their progeny phenotypes; *hacc_prog*: genotypes and phenotypes for high accuracy animals plus their progeny phenotypes

To check the quality of the IP and ${\text{ACC}}_{\text{IP}}$ for validation animals, we calculated the Pearson correlation between GEBV (obtained when included in the ssGBLUP) and IP (obtained when excluded), as well as between ${\text{ACC}}_{\text{GEBV}}$ and ${\text{ACC}}_{\text{IP}}$ (in the same two situations). Furthermore, a regression model was fitted as ${\text{ACC}}_{\text{GEBV}}={b}_{0}+{b}_{1}{\text{ACC}}_{\text{IP}}$ to investigate the presence of scale differences and dispersion in ${\text{ACC}}_{\text{IP}}$ calculation. Finally, we calculated the average and maximum absolute differences between ${\text{ACC}}_{\text{GEBV}}$ and ${\text{ACC}}_{\text{IP}}$ for all scenarios. All the analyses were performed using the BLUPF90 family of programs [27], after the modifications described in the implementation section, on a Linux server (x86_64) equipped with Intel Xeon E5-2470 2.30 GHz processors with 16 cores.

## Results and discussion

### IP and accuracy of IP

Correlations between GEBV and IP for post-weaning gain were ≥ 0.99 when 10K or more genotyped animals were used to backsolve SNP effects. With 5K and 2K, the correlations were 0.97 and 0.89, respectively. Previous studies have shown that IP can be safely obtained when using all genotyped animals with the APY algorithm, or using only a subset of the genotyped animals. However, when using only a subset of animals, the GEBV and genotypes used to backsolve SNP effects should come from the complete ssGBLUP evaluation using all available animals [1, 2, 22].

**Fig. 1:**
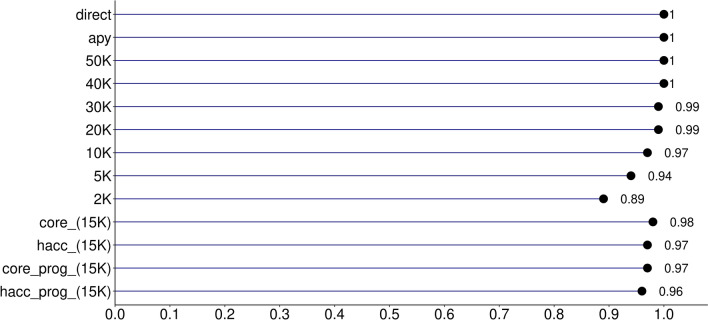
Correlation between ${\text{ACC}}_{\text{GEBV}}$ and ${\text{ACC}}_{\text{IP}}$. *direct*: all genotyped animals (N = 54,533) and phenotypes with direct ${\textbf{G}}^{-{\bf{1}}}$; *apy*: all genotyped animals (N = 54,533) and phenotypes with APY ${\textbf{G}}^{-{\bf{1}}}$; *50K-2K*: all phenotypes and decreasing the number of genotyped animals from 50K to 2K; *core*: genotypes for core animals only (N = 15K) and all phenotypes; *hacc*: genotypes for high accuracy animals only (N = 15K) and all phenotypes; *core_prog*: genotypes and phenotypes for core animals plus their progeny phenotypes; *hacc_prog*: genotypes and phenotypes for high accuracy animals plus their progeny phenotypes

**Fig. 2:**
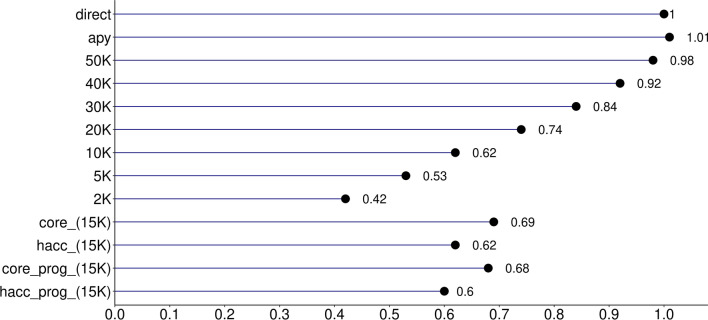
Regression coefficient (${b}_{1}$) of ${\text{the regression of ACC}}_{\text{GEBV}}$ on ${\text{ACC}}_{\text{IP}}$. *direct*: all genotyped animals (N = 54,533) and phenotypes with direct ${\textbf{G}}^{-{\bf{1}}}$; *apy*: all genotyped animals (N = 54,533) and phenotypes with APY ${\textbf{G}}^{-{\bf{1}}}$; *50K-2K*: all phenotypes and decreasing the number of genotyped animals from 50K to 2K; *core*: genotypes for core animals only (N = 15K) and all phenotypes; *hacc*: genotypes for high accuracy animals only (N = 15K) and all phenotypes; *core_prog*: genotypes and phenotypes for core animals plus their progeny phenotypes; *hacc_prog*: genotypes and phenotypes for high accuracy animals plus their progeny phenotypes

**Fig. 3:**
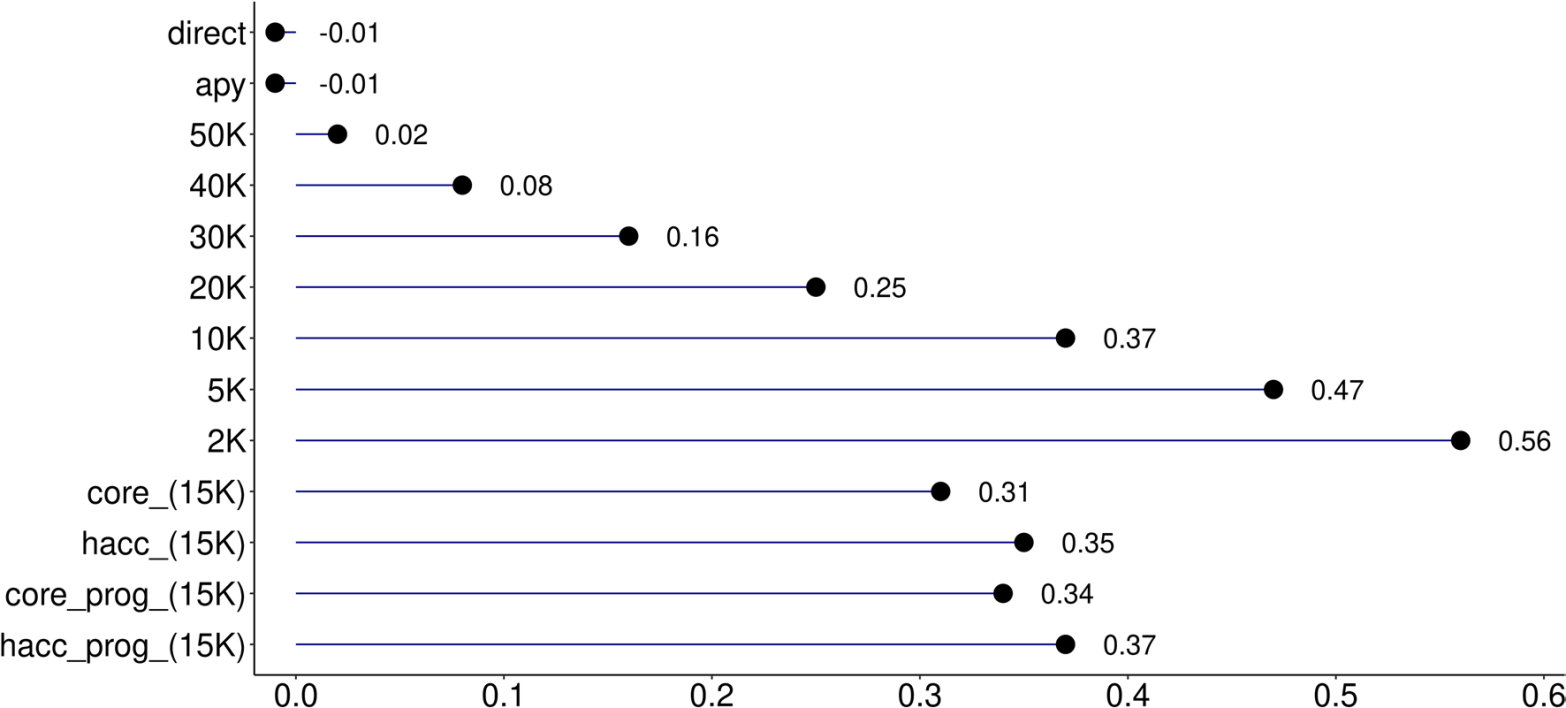
Intercept (${b}_{0}$) of the regression of ${\text{ACC}}_{\text{GEBV}}$ on ${\text{ACC}}_{\text{IP}}$. *direct*: all genotyped animals (N = 54,533) and phenotypes with direct ${\textbf{G}}^{-{\bf{1}}}$; *apy*: all genotyped animals (N = 54,533) and phenotypes with APY ${\textbf{G}}^{-{\bf{1}}}$; *50K-2K*: all phenotypes and decreasing the number of genotyped animals from 50K to 2K; *core*: genotypes for core animals only (N = 15K) and all phenotypes; *hacc*: genotypes for high accuracy animals only (N = 15K) and all phenotypes; *core_prog*: genotypes and phenotypes for core animals plus their progeny phenotypes; *hacc_prog*: genotypes and phenotypes for high accuracy animals plus their progeny phenotypes

The quality of the IP accuracies was evaluated based on the correlation between ${\text{ACC}}_{\text{GEBV}}$ and ${\text{ACC}}_{\text{IP}}$ and the intercept (${b}_{0}$), and the regression coefficient (${b}_{1}$) of ${\text{ACC}}_{\text{GEBV}}$ on ${\text{ACC}}_{\text{IP}}$ (Figs. 1, 2, 3). In the scenario where all genotyped animals and data were used to compute GEBV and PEC (*direct*), the correlation between ${\text{ACC}}_{\text{GEBV}}$ and ${\text{ACC}}_{\text{IP}}$ for the validation animals was 0.99, the intercept was − 0.01, and the regression coefficient was 1.00. In addition, the average and standard deviation of ${\text{ACC}}_{\text{GEBV}}$ and ${\text{ACC}}_{\text{IP}}$ in *direct* and *apy* were similar (Table 2). This shows that the implementation of accuracy for indirect predictions was successful. Using APY ${\textbf{G}}^{-{\bf{1}}}$ instead of the direct inversion of $\mathbf{G}$ did not change ${b}_{0}$ and the correlation between ${\text{ACC}}_{\text{GEBV}}$ and ${\text{ACC}}_{\text{IP}}$, but ${b}_{1}$ moved to 1.01, which is deemed negligible.

**Table 2:** Descriptive statistics for ${\text{ACC}}_{\text{GEBV}}$ and ${\text{ACC}}_{\text{IP}}$ for all scenarios and datasets

Scenario	Average	Min	Max	Standard deviation	ABS difference^a^	
					Average	Max
GEBV acc	0.73	0.27	0.82	0.03	NA	NA
direct	0.73	0.28	0.82	0.03	0.00	0.02
apy	0.74	0.28	0.82	0.03	0.01	0.03
50K	0.73	0.26	0.82	0.03	0.00	0.02
40K	0.71	0.21	0.8	0.03	0.02	0.08
30K	0.68	0.1	0.79	0.04	0.05	0.20
20K	0.64	0	0.76	0.04	0.09	0.34
10K	0.57	0	0.71	0.05	0.16	0.41
5K	0.5	0	0.67	0.05	0.23	0.48
2K	0.41	0	0.62	0.06	0.32	0.65
core (15K)	0.61	0	0.74	0.04	0.12	0.34
hacc (15K)	0.62	0	0.76	0.05	0.11	0.34
core_prog (15K)	0.57	0	0.7	0.04	0.16	0.41
hacc_prog (15K)	0.61	0	0.75	0.05	0.13	0.34

^a^ABS difference: absolute difference between ${\text{ACC}}_{\text{GEBV}}$ and ${\text{ACC}}_{\text{IP}}$

*direct*: all genotyped animals (N = 54,533) and phenotypes with direct ${\textbf{G}}^{-{\bf{1}}}$; *apy*: all genotyped animals (N = 54,533) and phenotypes with APY ${\textbf{G}}^{-{\bf{1}}}$; *50K-2K*: all phenotypes and decreasing the number of genotyped animals from 50K to 2K; *core*: genotypes for core animals only (N = 15K) and all phenotypes; *hacc*: genotypes for high accuracy animals only (N = 15K) and all phenotypes; *core_prog*: genotypes and phenotypes for core animals plus their progeny phenotypes; *hacc_prog*: genotypes and phenotypes for high accuracy animals plus their progeny phenotypes

By default, the BLUPF90 programs uses a small proportion of ${\mathbf{A}}_{22}$ (5%) to make $\mathbf{G}$ invertible in a process called blending [6]. We used the scenario *direct* to test larger proportions of blending and see the impact that they would have on ${\text{ACC}}_{\text{IP}}$. Although with higher blending proportions, (10–30%), the correlations between ${\text{ACC}}_{\text{GEBV}}$ and ${\text{ACC}}_{\text{IP}}$ were ≥ 0.99, the regression coefficient (${b}_{1}$) decreased, being as low as 0.86 when blending was up to 30% of ${\mathbf{A}}_{22}$ (Table 3). Ben Zaabza et al. [29] pointed out the importance of accounting for the residual polygenic effect when its proportion exceeds 20%, and our results show that while our current formulas and implementation are robust for smaller blending proportions, fine tuning is needed to account for higher proportions of blending or when a residual polygenic effect is explicitly included in the model.

**Table 3:** Correlation and regression coefficients for ${\text{ACC}}_{\text{GEBV}}$ and ${\text{ACC}}_{\text{IP}}$ for the *direct* scenario with different blending proportions

Scenario	Blending %	Correlation	b0	b1
direct	5	1.00	− 0.01	1.00
direct_10	10	1.00	− 0.01	0.98
direct_20	20	1.00	− 0.01	0.92
direct_30	30	1.00	− 0.01	0.86

The computing requirements for BLUPF90, POSTGSF90, and PREDF90 for the *direct* and *apy* scenarios are in Table 4. The total time for PREDF90 to calculate IP and ${\text{ACC}}_{\text{IP}}$ for the 5467 validation animals was approximately 30 min of which more than 99% was to calculate the accuracies (Eq. (19)). Although the memory for PREDF90 was the same in *direct* and *apy* because of the equal number of validation animals to compute IP, the memory needed for BLUPF90 and POSTGSf90 was about 10 GB more in the *apy* scenario. This is because a few extra temporary matrices and vectors are needed in APY. One expects APY to considerably reduce memory usage in BLUPF90 and POSTGSF90, which is true with the block implementation of APY adapted to the preconditioned conjugate gradient algorithm [30, 31]. When the inverse of the MME is involved such as in the computation of PEC, or variance components estimation, a full matrix with the dimension of the number of genotyped animals should be allocated to receive the elements of APY ${\textbf{G}}^{-{\bf{1}}}$, making the sparsity not exploitable, as shown previously by Junqueira et al. [32]. In addition, the authors point out that the creation of “fill in” elements may increase the amount of computing time necessary in the sparse inversion.

**Table 4:** Peak memory requirements for each scenario

Scenarios	Peak memory requirement (GB)^a^		
	BLUPF90	POSTGSF90	PREDF90
direct	195.60	228	11.6
apy	208.50	238	11.6
50K	182.75	211	11.6
40K	103.60	157	11.6
30K	69.40	113	11.6
20K	26.69	78	11.6
10K	5.65	52	11.6
5K	2.50	42	11.6
2K	0.63	38	11.6
core (15K)	18.50	63	11.6
hacc (15K)	17.90	63	11.6
core_prog (15K)	17.90	63	11.6
hacc_prog (15K)	18.20	63	11.6

^a^Real/resident memory (RSS); Linux server (x86_64) equipped with Intel Xeon E5-2470 2.30 GHz processors with 16 cores

*direct*: all genotyped animals (N = 54,533) and phenotypes with direct ${\textbf{G}}^{-{\bf{1}}}$; *apy*: all genotyped animals (N = 54,533) and phenotypes with APY ${\textbf{G}}^{-{\bf{1}}}$*; 50K-2K*: all phenotypes and decreasing the number of genotyped animals from 50K to 2K; *core*: genotypes for core animals only (N = 15K) and all phenotypes; *hacc*: genotypes for high accuracy animals only (N = 15K) and all phenotypes; *core_prog*: genotypes and phenotypes for core animals plus their progeny phenotypes; *hacc_prog*: genotypes and phenotypes for high accuracy animals plus their progeny phenotypes

When trying to reduce computing resources by cutting down the number of genotyped animals used in the computation of PEC from 50K to 2K, correlations between ${\text{ACC}}_{\text{GEBV}}$ and ${\text{ACC}}_{\text{IP}}$ were still 0.99 with as few as 20K genotyped animals (Fig. 1). Even when correlations between accuracies are high, we need to make sure that the ${\text{ACC}}_{\text{IP}}$ is unbiased, and that it is on the same scale as the ${\text{ACC}}_{\text{GEBV}}$. This will ensure that IP and its accuracy can be used as interim evaluations or replacements for GEBV if the number of genotyped animals becomes extremely large and it is desirable not to have young animals in the evaluation. For all the scenarios, the regression coefficient (${b}_{1}$) of ${\text{ACC}}_{\text{GEBV}}$ on ${\text{ACC}}_{\text{IP}}$ was used to evaluate dispersion and the intercept (${b}_{0}$) was used to check the scale. If there is no dispersion, ${b}_{1}$ equals 1, and deviations from one indicate either under or overestimation of ${\text{ACC}}_{\text{IP}}$. Regression coefficient and intercept for each scenario are presented in Figs. 2 and 3. No bias or scale differences were found when all genotyped animals (except for the validation) were used to calculate PEC in the scenarios *direct* and *apy*. However, reducing the number of genotyped animals to 20K increased inflation, as ${b}_{1}$ dropped from 0.98 to 0.74 (Fig. 2). At the same time, bias increased from 0.02 to 0.25 (Fig. 3), with a shift towards underestimation. Although an ad-hoc scaling factor based on tests with smaller datasets can correct the overall underestimation or overestimation, it is more difficult to reduce the inflation.

Using the number of eigenvalues explaining 99% of the variance of $\mathbf{G}$ (i.e., 15K) as the number of genotyped animals to obtain PEC resulted in a correlation of 0.98. Scenario *hacc* also had 15K core animals, but these were chosen based on high BLUP accuracy, and the correlation still reached 0.97. Correlations of 0.97 and 0.96 were found for the *hacc_prog* and *core_prog* scenarios, respectively, although the pedigree information was halved, and phenotypes reduced. As those 15K core animals are expected to represent most of the chromosome segments segregating in this Angus population, correlations remained high although only 25% of the available genotyped animals were used. The fact that correlations were not higher than 0.99 when using 15K core animals can be explained by the possible collinearity existing among those animals, thus slightly more animals would be required to reach a correlation higher than 0.99 [3]. In spite of the high correlations, using less data resulted in underestimation of ${\text{ACC}}_{\text{IP}}$, which was expected because less data leads to higher prediction error and consequently lower accuracy. In the current study, average ${\text{ACC}}_{\text{IP}}$ for the scenarios with 15K genotyped animals to compute PEC ranged from 0.57 to 0.62 and the regression coefficient ranged from 0.60 to 0.69. The memory requirement for BLUPF90 and POSTGSF90 when using 15K genotyped animals was on average 10 and 28% of that when using all available genotyped animals because of the much smaller number of elements in the coefficient matrix of ssGBLUP between the two scenarios.

As the number of genotyped animals in the PEC computations decreased further, ${\text{ACC}}_{\text{IP}}$ were underestimated and the difference in scale between ${\text{ACC}}_{\text{IP}}$ and ${\text{ACC}}_{\text{GEBV}}$ increased. For instance, ${b}_{1}$ was as low as 0.42 and ${b}_{0}$ as high as 0.56 when using 2K genotyped animals. Typically, when ${b}_{1}$ is lower than 1, the conclusion is that the predictions are overestimated; however, this is true when ${b}_{0}$ is close to 0. When 30K or less genotyped animals were used to compute PEC, the intercept was not 0 and although ${b}_{1}$ was lower than 1, ${\text{ACC}}_{\text{IP}}$ were underestimated rather than overestimated (Table 2). Differences between ${\text{ACC}}_{\text{IP}}$ and ${\text{ACC}}_{\text{GEBV}}$ can also be seen in the average and maximum absolute changes (Table 2). Following a similar pattern, as the number of animals decreased, average and maximum changes increased. For instance, average and maximum changes were 0.00 and 0.02 when 50K animals were used but increased to 0.32 and 0.64 when only 2K animals were used. For the scenarios with 40K or more animals, average and maximum accuracy differences were at most 0.02 and 0.08, respectively.

Although we were able to successfully approximate SNP PEC and obtain reasonable values of ${\text{ACC}}_{\text{IP}}$ with 50K or 40K genotyped animals, ${\text{ACC}}_{\text{IP}}$ deteriorated with fewer genotyped animals. As the number of genotyped animals decrease, the contributions due to the ${\mathbf{G}}^{-{1}}-{\mathbf{A}}_{22}^{-{1}}$ block of MME decrease and the approximation of PEC becomes poor, resulting in underestimated IP accuracies. Even when the number of animals in the pedigree and the number of records remained constant in most of the scenarios (Table 1), the changes in ${\text{ACC}}_{\text{IP}}$ depended on the number of genotyped animals used to compute SNP PEC. Furthermore, using only own and progeny records, did not result in increased dispersion compared to using complete data and pedigree information (*core* vs *core_prog* and *hacc* vs *hacc_prog* scenarios in Fig. 3). It is worth noting that the number of records and animals in the pedigree was nearly halved between the *core* and *core_prog* scenarios. This indicates that including a sufficient number of genotyped animals with own phenotypes, and adding their phenotyped progeny are enough to account for the contributions due to phenotypes and pedigrees as well as ${\mathbf{G}}^{-1}-{\mathbf{A}}_{22}^{-1}$ and to obtain reasonable SNP PEC for IP accuracy. For future research with larger datasets, groups of genotyped animals with many phenotyped progeny could be a good target when trying to reduce computation costs in obtaining SNP PEC.

With 40K to 50K genotyped animals, it was possible to obtain ${\text{ACC}}_{\text{IP}}$ without a severe dispersion, which represents 67 and 83% of the total genotyped animals. In addition, our results suggest that using as few as 15K genotyped animals, or the number of eigenvalues explaining 99% of the variance of $\mathbf{G}$, can yield correlations between ${\text{ACC}}_{\text{IP}}$ and ${\text{ACC}}_{\text{GEBV}}$ that are as high as 0.98. However, it is important to note that with a smaller number of animals, even when blending and tuning parameters were considered, there was still a scaling issue and ${\text{ACC}}_{\text{IP}}$ were underestimated. Using SNP PEC from a SNP-BLUP model, Erbe et al. [13] found that the composition of the reference population affected the quality of the final approximation of GEBV accuracies from the Interbull standardized genomic reliability model, and pointed out that under ssGBLUP, the definition of such a reference population is not as clear as in the multi-step procedure, which would require further investigation to define which animals should be included in PEC computations from ssGBLUP.

The inversion of the LHS to obtain SNP PEC from a ssGBLUP model is a computationally demanding step in the process of calculating accuracies for IP; therefore, reducing the overall size of the MME by reducing the number of genotyped animals is of interest for routine applications. Compared to the approach presented by Liu et al. [12] for a SNP-BLUP model, obtaining SNP PEC from ssGBLUP may be challenging because it depends on the number of animals rather than the number of SNPs included in the system of equations, therefore reducing the number of genotyped animals for PEC computations is critical. Methods to approximate PEC could be likewise helpful.

More research is needed to investigate whether SNP PEC computed from a smaller subset of genotyped animals can be used to approximate ${\text{ACC}}_{\text{IP}}$ with a larger number of genotyped animals and to account for large proportions of blending or the residual polygenic effect. Such tests could be hard to accomplish because obtaining ${\text{ACC}}_{\text{GEBV}}$ based on PEV as a benchmark is not feasible for large datasets, although approximations could be used. In addition, to be able to use smaller subsets of animals, fine tuning is still needed to refine the methods and to define which animals should be used in PEC computations to avoid biases on IP accuracy. Finally, although it is outside of the scope of this study, since SNP PEC accounts for the genomic contributions from ssGBLUP MME, a combination of our approach with existing PEV approximations may be useful to obtain GEBV accuracies for large-scale ssGBLUP evaluations.

## Conclusions

Indirect prediction accuracy can be successfully obtained by computing SNP PEC from the single-step mixed model equations using direct inversion of the genomic relationship matrix or by the APY algorithm. With at least 40K out of 60K genotyped animals included in PEC calculations, robust indirect prediction accuracies can be obtained without dispersion issues. To reduce the computational costs of inverting the left-hand-side of the mixed model equations, SNP PEC can be approximated by using a smaller subset of the genotyped animals. This yields high correlations, but fine tuning is still required to scale accuracies of indirect predictions up to accuracies of GEBV. Using genotyped sires with phenotyped progeny could help mitigate this issue. Further studies are needed to develop SNP PEC approximations and extend it to large-scale genomic data.

## Data Availability

The data used in this study were provided by the American Angus Association and are used under license for the current study, so they are not publicly available, and restrictions apply to their availability. Modified BLUPF90, POSTGSF90 (to save PEC of SNP effects), and PREDF90 (to compute accuracy of IP) are freely available for research purposes on up to 25,000 genotyped animals at http://nce.ads.uga.edu/html/projects/programs/.
