# Correction: Theoretical accuracy for indirect predictions based on SNP effects from single-step GBLUP

**DOI:** 10.1186/s12711-023-00799-x

**Published:** 2023-04-17

**Authors:** Andre Garcia, Ignacio Aguilar, Andres Legarra, Shogo Tsuruta, Ignacy Misztal, Daniela Lourenco

**Affiliations:** 1 Department of Animal and Dairy Science, University of Georgia, Athens, GA 30602 USA; 2 Instituto Nacional de Investigación Agropecuaria (INIA), 11500 Montevideo, Uruguay; 3 UMR GenPhySE, INRA Toulouse, BP52626, 31326 Castanet Tolosan, France

**Correction: Genetics Selection Evolution (2022) 54:66** **https://doi.org/10.1186/s12711-022-00752-4**

After publication of this work [1], we noticed that there was an error in Eqs. (14) to (19) presented on page 4 of the paper, where we refer to the term $\mathrm{var}(\widehat{\mathbf{a}})$ as the prediction error covariance (PEC) of SNP effects which is not correct.

The correct equations should be:

14 $${\mathbf{var}}\left(\widehat{\mathbf{a}}\right)\text{ = var}\left(\left({1}-{\alpha }\right){{b}}{\frac{1}{2\sum {\mathrm{p}}_{i}\left(1-{\mathrm{p}}_{i}\right)}\mathbf{Z}}{\boldsymbol{^{\prime}}}{\mathbf{G}}^{-{1}}\widehat{\mathbf{u}}\right)$$

15 $$=\left({1}-\alpha \right){{b}}{\frac{1}{2\sum {\mathrm{p}}_{i}\left(1-{\mathrm{p}}_{i}\right)}\mathbf{Z}}{\mathbf{^{\prime}}}{\mathbf{G}}^{-{\boldsymbol{1}}}\mathrm{var}(\widehat{\mathbf{u}}){\left(\left(1-\alpha \right){{b}}\frac{1}{2\sum {\mathrm{p}}_{i}\left(1-{\mathrm{p}}_{i}\right)}{\mathbf{Z}}{\boldsymbol{^{\prime}}}{\mathbf{G}}^{-{\boldsymbol1}}\right)}^{^{\prime}}$$

16 $$=\left({1}-\mathrm{\alpha }\right){{b}}{\frac{1}{2\sum {\mathrm{p}}_{i}\left(1-{\mathrm{p}}_{i}\right)}\mathbf{Z}}{\mathbf{^{\prime}}}{\mathbf{G}}^{-{\boldsymbol{1}}}\left(\mathrm{var}\left(\mathbf{u}\right)-\mathrm{var}\left(\widehat{\mathbf{u}}-\mathbf{u}\right)\right){\mathbf{G}}^{-{\boldsymbol{1}}}{\mathbf{Z}}\frac{1}{2\sum {\mathrm{p}}_{i}\left(1-{\mathrm{p}}_{i}\right)}{{b}}\left({1}-{\alpha }\right).$$

Then,

17 $${\text{var}}\left(\widehat{\mathbf{a}}\right)= \text{ } \left({1}-{\alpha }\right){b}{\frac{1}{2\sum {\mathrm{p}}_{i}\left(1-{\mathrm{p}}_{i}\right)}\mathbf{Z}}{\boldsymbol{^{\prime}}}{\mathbf{G}}^{-{\boldsymbol{1}}}\left({\mathbf{G}}{\upsigma}_{\text{u}}^{2}-{\mathbf{C}}^{{\mathrm{u}}_{2}{\mathrm{u}}_{2}}\right){\mathbf{G}}^{-{\boldsymbol{1}}}{\mathbf{Z}}\frac{1}{2\sum {\mathrm{p}}_{i}\left(1-{\mathrm{p}}_{i}\right)}b\left({1}-{\alpha }\right).$$

Therefore,

18 $${\text{var}}\left(\widehat{\mathbf{a}}\right)= \text{ } \left({1}-\alpha \right){{b}}{\frac{1}{2\sum {\mathrm{p}}_{i}\left(1-{\mathrm{p}}_{i}\right)}(\mathbf{Z}}{\boldsymbol{^{\prime}}}{\mathbf{G}}^{-{\boldsymbol{1}}}\mathbf{Z}{\upsigma}_{\text{u}}^{2} \, -{\mathbf{Z}}{\boldsymbol{^{\prime}}{\mathbf{G}}^{-{\boldsymbol{1}}}{\mathbf{C}}^{{\mathrm{u}}_{2}{\mathrm{u}}_{2}}{\mathbf{G}}^{-{\boldsymbol1}}}\mathbf{Z)}\frac{1}{2\sum {\mathrm{p}}_{i}\left(1-{\mathrm{p}}_{i}\right)}b\left({1}-\alpha \right).$$

19 $${\mathrm{ACC}}_{{\mathrm{IP}}_{j}} \, \text{=}\sqrt{\frac{{\mathbf{z}}_{{j}}{\text{var}}\left(\widehat{\mathbf{a}}\right){\mathbf{z}}_{{j}}^{\prime}}{{\upsigma}_{\text{u}}^{2}}}.$$

Please note that the equations are correctly programmed in the software used for analyses, and the corrections above do not affect the end results presented in the manuscript.

We thank Dr. Matias Berman from The University of Georgia and Dr. Jeremie Vandenplas from Wageningen University for their interest and suggestions to correct the manuscript.
